# Supplementary material for: Dissecting clinical heterogeneity of bipolar disorder using multiple polygenic risk scores
Source: Transl Psychiatry. 2020 Sep 18;10:314. doi: 10.1038/s41398-020-00996-y (PMC7501305; doi:10.1038/s41398-020-00996-y)
Supplement: Supplementary file 1 — Supplemental Material [file 41398_2020_996_MOESM1_ESM.docx]

| **Supplementary Table 1:** Sub-phenotype definitions assessed in Mayo and GAIN datasets | | |
| --- | --- | --- |
| **Sub-phenotype** | **Study** | **Definition** |
| BD Type | Mayo | Assessed via SCID |
|  |  |  |
|  | GAIN | Only BD-I cases were ascertained |
|  |  |  |
| Psychosis | Mayo | Ascertained using SCID history of psychosis during mania and/or depression |
|  |  |  |
|  |  | *40 cases experienced psychosis during both mania and depression (assigned to manic psychosis group)* |
|  | GAIN | History of psychosis assessed in DIGS. |
|  |  |  |
|  |  | *Psychosis is assumed to be during mania because that is most prevalent in BD-I* |
| Rapid Cycling | Mayo | Ascertained using SCID history of rapid cycling (> 3 episodes in a year) |
|  |  |  |
|  | GAIN | Asked differently from DIGS version |
|  |  | DIGSv2.0 – “Have you had at least four episodes of mood disorder within a one-year period?” |
|  |  | DIGSv3.0 – “Have you ever had a year when you had several different manic, hypomanic, depressive, or mixed episodes?” combined with # of affective episodes in 1 year (> 3 episodes) |
|  |  | DIGSv4.0 – “Have you ever had a year when you had several different manic, hypomanic, depressive, or mixed episodes?” and how many (> 3 is rapid) |
| Age-of-onset | Mayo | Ascertained using patient questionnaire on age of diagnosis of BD collected in ranges (19 or younger, 20 to 49, 50 to 64, 65 or older) |
|  |  |  |
|  |  | *Dichotomized as early (19 or younger) or non-early (all other age ranges)* |
|  | GAIN | Assessed age of first major depression, mania, and hypomania. |
|  |  |  |
|  |  | *Early onset is minimum age of above dichotomized (< 19)* |
| Attempted suicide | Mayo | Ascertained using SCID whether they have attempted suicide. Ascertained via patient questionnaire how many suicide attempts. |
|  |  |  |
|  | GAIN | DIGS asked patients whether they have attempted suicide |
|  |  |  |

| **Supplementary Table 2:** Summaries of the PRSs used in the study. N_eff = effective sample size; r_g = Genetic correlation from LDSC with BD-PGC2 summary statistics (Stahl et al. 2019); p_rg = p-value for genetic correlation; Red font denotes where a study did not fit our inclusion criteria | | | | | | | | | |
| --- | --- | --- | --- | --- | --- | --- | --- | --- | --- |
| **PRS_NAME** | **Trait** | **N_eff** | **r_g** | **p_rg** | **Ancestry** | **First.Author** | **Year** | **Journal** | **Link** |
| BD_PGC2 | Bipolar disorder | 50619 | 1 | 0.00E+00 | EUR | E Stahl | 2019 | Nature Genetics | https://www.nature.com/articles/s41588-019-0397-8 |
| SCZ_PGC2 | Schizophrenia | 55743 | 0.717 | 4.00E-289 | EUR | S Ripke | 2014 | Nature | https://www.nature.com/articles/nature13595 |
| MDD_PGCUKB | Major Depressive Disorder | 342408 | 0.356 | 4.60E-49 | EUR | D Howard | 2019 | Nature Neuroscience | https://www.ncbi.nlm.nih.gov/pubmed/30718901 |
| EA_SSGAC | Educational Attainment | 1100000 | 0.187 | 3.60E-20 | Mostly EUR | J Lee | 2018 | Nature Genetics | <https://www.ncbi.nlm.nih.gov/pubmed/30038396> |
| RISK_UKB | Risk-taking behavior | 116255 | 0.305 | 4.20E-20 | EUR | R Strawbridge | 2018 | Transl Psychiatry | https://www.nature.com/articles/s41398-017-0079-1 |
| AUD_MVPTE | Alcohol use disorder (ICD defined) | 274424 | 0.35 | 2.10E-18 | Diverse | H Kranzler | 2019 | Nature Comm | https://www.nature.com/articles/s41467-019-09480-9 |
| ANXIETY_UKBiPSYCHANGST | Anxiety disorders | 60568 | 0.306 | 1.37E-14 | EUR | K Purves | 2019 | Molecular Psychiatry | https://www.ncbi.nlm.nih.gov/pubmed/31748690 |
| OCD_PGC | Obsessive compulsive disorder | **3890** | 0.309 | 3.90E-07 | EUR | IOCDF-GC | 2018 | Molecular Psychiatry | https://www.ncbi.nlm.nih.gov/pubmed/28761083 |
| AN_PGC2 | Anorexia Nervosa | 26021 | -0.177 | 6.50E-07 | EUR | H Watson | 2019 | Nature Genetics | https://www.nature.com/articles/s41588-019-0439-2 |
| SWB_SSGAC | Subjective Well-being | 298420 | -0.206 | 7.10E-07 | Mostly EUR | A Okbay | 2016 | Nature Genetics | https://www.ncbi.nlm.nih.gov/pubmed/27089181 |
| ANHEDONIA_UKB | Anhedonia | 375275 | 0.133 | 1.52E-05 | EUR | J Ward | 2019 | Transl Psychiatry | <https://www.nature.com/articles/s41398-019-0635-y> |
| NEUROTICISM_SSGAC | Neuroticism | 168105 | 0.121 | 3.00E-04 | EUR | P Turley | 2018 | Nature Genetics | https://www.ncbi.nlm.nih.gov/pubmed/29292387 |
| ADHD_PGC | ADHD | 25653 | 0.135 | 3.00E-04 | EUR+Chinese | D Demontis | 2019 | Nature Genetics | https://www.ncbi.nlm.nih.gov/pubmed/30478444 |
| BMI_UKBB | Body Mass Index | 359983 | -0.071 | 1.00E-03 | EUR | n/a | n/a | n/a | http://www.nealelab.is/uk-biobank |
| PTSD_PGC2 | Post-traumatic stress disorder | 32428 | 0.19 | 1.00E-03 | Diverse | C Nievergelt | 2019 | Nature Comm | <https://www.nature.com/articles/s41467-019-12576-w> |
| INSOMNIA_UKB | Insomnia (self-report) | 453379 | 0.055 | **0.084** | EUR | J Lane | 2019 | Nature Genetics | https://www.nature.com/articles/s41588-019-0361-7 |
| AUDITC_MVPTE | Alcohol consumption | 274424 | -0.003 | **0.95** | Diverse | H Kranzler | 2019 | Nature Comm | https://www.nature.com/articles/s41467-019-09480-8 |

| **Supplementary Table 3:** Full results for each PRS tested with psychosis. Marginal = model with only one PRS at a time; Joint = model including significant PRSs; X2.5 = lower 95% CI; X97.5 = upper 95% CI | | | | | | | | | | | | | |
| --- | --- | --- | --- | --- | --- | --- | --- | --- | --- | --- | --- | --- | --- |
| **PRS** | **Name** | **OR.marginal** | **X2.5...marginal** | **X97.5...marginal** | **r2.marginal** | **pval.marginal** | **OR.joint** | **X2.5...joint** | **X97.5...joint** | **r2.joint** | **pval.joint** | **P.joint.joint** | **R2.all.joint** |
| SCZ_PGC2 | SCZ | 1.30 | 1.15 | 1.48 | 1.22% | 3.5E-05 | 1.34 | 1.18 | 1.52 | 1.44% | 8.0E-06 | 8.0E-08 | 2.6E-02 |
| ANHEDONIA_UKB | Anhedonia | 0.87 | 0.79 | 0.95 | 0.59% | 3.2E-03 | 0.85 | 0.77 | 0.93 | 0.77% | 8.6E-04 | 8.0E-08 | 2.6E-02 |
| BMI_UKBB | BMI | 0.87 | 0.79 | 0.95 | 0.57% | 3.6E-03 | 0.89 | 0.81 | 0.98 | 0.33% | 2.3E-02 | 8.0E-08 | 2.6E-02 |
| BD_PGC2 | BD | 1.21 | 1.04 | 1.42 | 0.40% | 1.3E-02 | NA | NA | NA | NA | NA | NA | NA |
| EA_SSGAC | EA | 1.12 | 1.02 | 1.24 | 0.35% | 2.0E-02 | NA | NA | NA | NA | NA | NA | NA |
| ADHD_PGC | ADHD | 0.90 | 0.81 | 0.99 | 0.29% | 3.2E-02 | NA | NA | NA | NA | NA | NA | NA |
| PTSD_PGC2 | PTSD | 0.90 | 0.80 | 1.01 | 0.20% | 6.5E-02 | NA | NA | NA | NA | NA | NA | NA |
| ANXIETY_UKBiPSYCHANGST | Anxiety | 0.95 | 0.85 | 1.06 | 0.01% | 3.5E-01 | NA | NA | NA | NA | NA | NA | NA |
| MDD_PGCUKB | MDD | 0.96 | 0.87 | 1.06 | 0.00% | 4.5E-01 | NA | NA | NA | NA | NA | NA | NA |
| NEUROTICISM_SSGAC | Neuroticism | 0.97 | 0.88 | 1.07 | 0.00% | 5.0E-01 | NA | NA | NA | NA | NA | NA | NA |
| AUD_MVPTE | AUD | 1.02 | 0.93 | 1.13 | 0.00% | 6.5E-01 | NA | NA | NA | NA | NA | NA | NA |
| AN_PGC2 | AN | 0.99 | 0.88 | 1.11 | 0.00% | 8.4E-01 | NA | NA | NA | NA | NA | NA | NA |
| RISK_UKB | Risk-taking | 0.99 | 0.90 | 1.10 | 0.00% | 8.7E-01 | NA | NA | NA | NA | NA | NA | NA |

| **Supplementary Table 4:** Full results for each PRS tested with early-onset BD. Marginal = model with only one PRS at a time; Joint = model including significant PRSs; X2.5 = lower 95% CI; X97.5 = upper 95% CI | | | | | | | | | | | | | |
| --- | --- | --- | --- | --- | --- | --- | --- | --- | --- | --- | --- | --- | --- |
| **PRS** | **Name** | **OR.marginal** | **X2.5...marginal** | **X97.5...marginal** | **r2.marginal** | **pval.marginal** | **OR.joint** | **X2.5...joint** | **X97.5...joint** | **r2.joint** | **pval.joint** | **P.joint.joint** | **R2.all.joint** |
| RISK_UKB | Risk-taking | 1.21 | 1.09 | 1.35 | 0.92% | 0.0005 | 1.20 | 1.08 | 1.34 | 0.83% | 0.0008 | 6.3E-05 | 1.5E-02 |
| ANHEDONIA_UKB | Anhedonia | 1.16 | 1.05 | 1.29 | 0.58% | 0.0047 | 1.15 | 1.04 | 1.27 | 0.49% | 0.0089 | 6.3E-05 | 1.5E-02 |
| ADHD_PGC | ADHD | 1.15 | 1.03 | 1.28 | 0.46% | 0.0102 | NA | NA | NA | NA | NA | NA | NA |
| PTSD_PGC2 | PTSD | 1.17 | 1.04 | 1.32 | 0.46% | 0.0107 | NA | NA | NA | NA | NA | NA | NA |
| BD_PGC2 | BD | 0.83 | 0.71 | 0.98 | 0.33% | 0.0278 | NA | NA | NA | NA | NA | NA | NA |
| AN_PGC2 | AN | 0.89 | 0.78 | 1.01 | 0.22% | 0.0622 | NA | NA | NA | NA | NA | NA | NA |
| EA_SSGAC | EA | 0.92 | 0.83 | 1.02 | 0.15% | 0.1040 | NA | NA | NA | NA | NA | NA | NA |
| ANXIETY_UKBiPSYCHANGST | Anxiety | 1.09 | 0.97 | 1.22 | 0.12% | 0.1336 | NA | NA | NA | NA | NA | NA | NA |
| MDD_PGCUKB | MDD | 1.06 | 0.96 | 1.18 | 0.05% | 0.2461 | NA | NA | NA | NA | NA | NA | NA |
| AUD_MVPTE | AUD | 0.94 | 0.85 | 1.05 | 0.04% | 0.2806 | NA | NA | NA | NA | NA | NA | NA |
| SCZ_PGC2 | SCZ | 0.95 | 0.84 | 1.09 | 0.00% | 0.4859 | NA | NA | NA | NA | NA | NA | NA |
| BMI_UKBB | BMI | 1.02 | 0.92 | 1.13 | 0.00% | 0.7284 | NA | NA | NA | NA | NA | NA | NA |
| SWB_SSGAC | SWB | 0.99 | 0.89 | 1.10 | 0.00% | 0.7936 | NA | NA | NA | NA | NA | NA | NA |
| NEUROTICISM_SSGAC | Neuroticism | 1.01 | 0.91 | 1.12 | 0.00% | 0.8462 | NA | NA | NA | NA | NA | NA | NA |

| **Supplementary Table 5:** Full results for each PRS tested with rapid cycling. Marginal = model with only one PRS at a time; Joint = model including significant PRSs; X2.5 = lower 95% CI; X97.5 = upper 95% CI | | | | | | | | | | | | | |
| --- | --- | --- | --- | --- | --- | --- | --- | --- | --- | --- | --- | --- | --- |
| **PRS** | **Name** | **OR.marginal** | **X2.5...marginal** | **X97.5...marginal** | **r2.marginal** | **pval.marginal** | **OR.joint** | **X2.5...joint** | **X97.5...joint** | **r2.joint** | **pval.joint** | **P.joint.joint** | **R2.all.joint** |
| MDD_PGCUKB | MDD | 1.23 | 1.11 | 1.36 | 1.22% | 0.00004 | 1.158 | 1.032 | 1.301 | 0.41% | 0.0129 | 4.67E-10 | 0.039 |
| PTSD_PGC2 | PTSD | 1.28 | 1.14 | 1.44 | 1.21% | 0.00004 | 1.217 | 1.078 | 1.375 | 0.71% | 0.0015 | 4.67E-10 | 0.039 |
| ADHD_PGC | ADHD | 1.23 | 1.11 | 1.36 | 1.14% | 0.00007 | 1.183 | 1.066 | 1.313 | 0.70% | 0.0016 | 4.67E-10 | 0.039 |
| ANXIETY_UKBiPSYCHANGST | Anxiety | 1.19 | 1.07 | 1.33 | 0.71% | 0.00145 | 1.068 | 0.944 | 1.210 | 0.03% | 0.2974 | 4.67E-10 | 0.039 |
| BD_PGC2 | BD | 0.80 | 0.68 | 0.93 | 0.56% | 0.00437 | 0.752 | 0.641 | 0.881 | 0.89% | 0.0004 | 4.67E-10 | 0.039 |
| NEUROTICISM_SSGAC | Neuroticism | 1.14 | 1.04 | 1.26 | 0.51% | 0.00609 | NA | NA | NA | NA | NA | NA | NA |
| ANHEDONIA_UKB | Anhedonia | 1.14 | 1.04 | 1.25 | 0.49% | 0.00736 | NA | NA | NA | NA | NA | NA | NA |
| EA_SSGAC | EA | 0.88 | 0.80 | 0.97 | 0.46% | 0.00917 | NA | NA | NA | NA | NA | NA | NA |
| BMI_UKBB | BMI | 1.13 | 1.03 | 1.24 | 0.42% | 0.01209 | NA | NA | NA | NA | NA | NA | NA |
| RISK_UKB | Risk-taking | 1.12 | 1.01 | 1.23 | 0.30% | 0.03057 | NA | NA | NA | NA | NA | NA | NA |
| AN_PGC2 | AN | 0.90 | 0.79 | 1.01 | 0.18% | 0.07769 | NA | NA | NA | NA | NA | NA | NA |
| SCZ_PGC2 | SCZ | 0.90 | 0.79 | 1.02 | 0.16% | 0.08843 | NA | NA | NA | NA | NA | NA | NA |
| AUD_MVPTE | AUD | 1.01 | 0.92 | 1.11 | 0.00% | 0.82606 | NA | NA | NA | NA | NA | NA | NA |
| SWB_SSGAC | SWB | 1.00 | 0.91 | 1.11 | 0.00% | 0.95416 | NA | NA | NA | NA | NA | NA | NA |

| **Supplementary Table 6:** Full results for each PRS tested with attempted suicide. Marginal = model with only one PRS at a time; Joint = model including significant PRSs; X2.5 = lower 95% CI; X97.5 = upper 95% CI | | | | | | | | | | | | | |
| --- | --- | --- | --- | --- | --- | --- | --- | --- | --- | --- | --- | --- | --- |
| **PRS** | **Name** | **OR.marginal** | **X2.5...marginal** | **X97.5...marginal** | **r2.marginal** | **pval.marginal** | **OR.joint** | **X2.5...joint** | **X97.5...joint** | **r2.joint** | **pval.joint** | **P.joint.joint** | **R2.all.joint** |
| MDD_PGCUKB | MDD | 1.26 | 1.15 | 1.39 | 1.57% | 0.000001 | 1.19 | 1.07 | 1.32 | 0.69% | 0.0011 | 2.66E-07 | 0.0229 |
| ANHEDONIA_UKB | Anhedonia | 1.22 | 1.12 | 1.34 | 1.25% | 0.000016 | 1.12 | 1.01 | 1.24 | 0.29% | 0.0265 | 2.66E-07 | 0.0229 |
| EA_SSGAC | EA | 0.87 | 0.79 | 0.96 | 0.53% | 0.003648 | 0.92 | 0.83 | 1.01 | 0.15% | 0.0862 | 2.66E-07 | 0.0229 |
| PTSD_PGC2 | PTSD | 1.16 | 1.04 | 1.30 | 0.44% | 0.007918 | NA | NA | NA | NA | NA | NA | NA |
| BD_PGC2 | BD | 0.83 | 0.72 | 0.96 | 0.37% | 0.013184 | NA | NA | NA | NA | NA | NA | NA |
| NEUROTICISM_SSGAC | Neuroticism | 1.12 | 1.02 | 1.23 | 0.37% | 0.013769 | NA | NA | NA | NA | NA | NA | NA |
| ANXIETY_UKBiPSYCHANGST | Anxiety | 1.11 | 1.00 | 1.23 | 0.24% | 0.040121 | NA | NA | NA | NA | NA | NA | NA |
| RISK_UKB | Risk-taking | 1.10 | 1.00 | 1.21 | 0.22% | 0.048111 | NA | NA | NA | NA | NA | NA | NA |
| ADHD_PGC | ADHD | 1.09 | 0.99 | 1.20 | 0.17% | 0.076918 | NA | NA | NA | NA | NA | NA | NA |
| SWB_SSGAC | SWB | 0.94 | 0.86 | 1.04 | 0.05% | 0.216049 | NA | NA | NA | NA | NA | NA | NA |
| AUD_MVPTE | AUD | 1.05 | 0.96 | 1.15 | 0.02% | 0.299444 | NA | NA | NA | NA | NA | NA | NA |
| BMI_UKBB | BMI | 1.05 | 0.96 | 1.15 | 0.02% | 0.315576 | NA | NA | NA | NA | NA | NA | NA |
| SCZ_PGC2 | SCZ | 0.94 | 0.84 | 1.06 | 0.01% | 0.334892 | NA | NA | NA | NA | NA | NA | NA |
| AN_PGC2 | AN | 0.99 | 0.88 | 1.11 | 0.00% | 0.824200 | NA | NA | NA | NA | NA | NA | NA |

**Supplementary Figure 1:** Correlations between the PRSs in the analyses. Correlations are computed in the combined GAIN + Mayo sample.


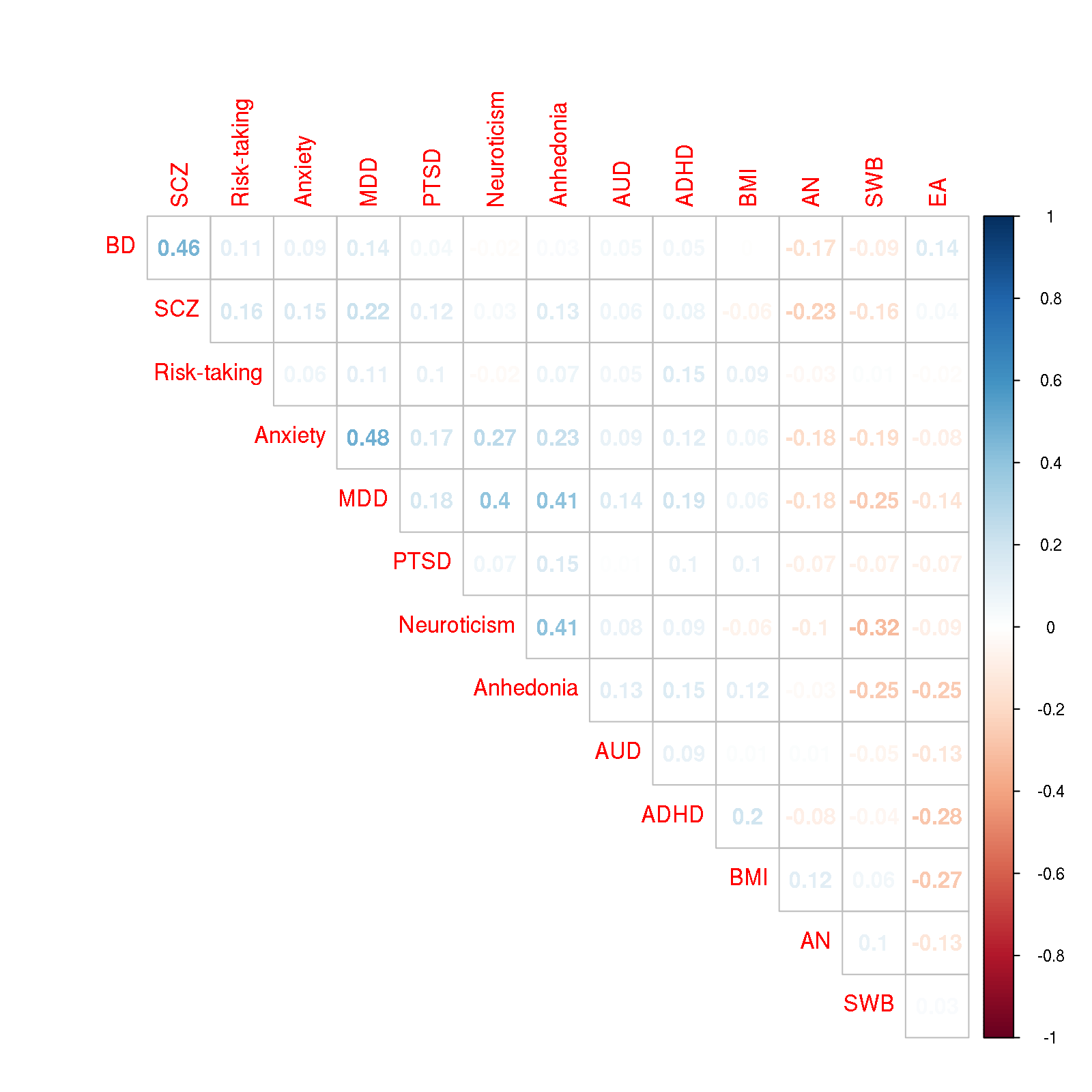


**Supplementary Data**

**PRS Data**. The dataset required to reproduce our analyses are provided in the supplementary material. The description of each variable is below:

Study = MAYO or GAIN

SEX: 1 = Female, 0 = Male

BD1vBD2: 1 = Bipolar Type I, 0 = Bipolar Type II

MPvNOP: 1 = Psychosis, 0 = No psychosis

EOvNOTEO: 1 = Early-onset BD (aoo < 18), 0 = Non-early-onset BD (aoo > 18)

RvNOTR: 1 = Rapid cycling, 0 = No rapid cycling history

Suicide_attempt: 1 = Yes, 0 = No

EV*: First four principal components (PCs) for ancestry

*.prs.pc: The final PRScs-derived PRSs for each trait
